# Genome assembly of *Melilotus officinalis* provides a new reference genome for functional genomics

**DOI:** 10.1186/s12863-024-01224-y

**Published:** 2024-04-18

**Authors:** Aoran Meng, Xinru Li, Zhiguang Li, Fuhong Miao, Lichao Ma, Shuo Li, Wenfei Sun, Jianwei Huang, Guofeng Yang

**Affiliations:** 1 Key Laboratory of National Forestry and Grassland Administration on Grassland Resources and Ecology in the Yellow River Delta, College of Grassland Science, Qingdao Agricultural University, 266109 Qingdao, China; 2 Berry Genomics Corporation, Beijing, China

**Keywords:** *Melilotus officinalis*, Genome, Assembly, PacBio, HiFi

## Abstract

**Background:**

Sweet yellow clover (*Melilotus officinalis*) is a diploid plant (2n = 16) that is native to Europe. It is an excellent legume forage. It can both fix nitrogen and serve as a medicine. A genome assembly of *Melilotus officinalis* that was collected from Best corporation in Beijing is available based on Nanopore sequencing. The genome of *Melilotus officinalis* was sequenced, assembled, and annotated.

**Results:**

The latest PacBio third generation HiFi assembly and sequencing strategies were used to produce a *Melilotus officinalis* genome assembly size of 1,066 Mbp, contig N50 = 5 Mbp, scaffold N50 = 130 Mbp, and complete benchmarking universal single-copy orthologs (BUSCOs) = 96.4%. This annotation produced 47,873 high-confidence gene models, which will substantially aid in our research on molecular breeding. A collinear analysis showed that *Melilotus officinalis* and *Medicago truncatula* shared conserved synteny. The expansion and contraction of gene families showed that *Melilotus officinalis* expanded by 565 gene families and shrank by 56 gene families. The contacted gene families were associated with response to stimulus, nucleotide binding, and small molecule binding. Thus, it is related to a family of genes associated with peptidase activity, which could lead to better stress tolerance in plants.

**Conclusions:**

In this study, the latest PacBio technology was used to assemble and sequence the genome of the *Melilotus officinalis* and annotate its protein-coding genes. These results will expand the genomic resources available for *Melilotus officinalis* and should assist in subsequent research on sweet yellow clover plants.

**Supplementary Information:**

The online version contains supplementary material available at 10.1186/s12863-024-01224-y.

## Background

Sweet yellow clover (*Melilotus officinalis*) is a legume that is native to Europe and widely distributed in North America, temperate Europe, the Mediterranean, subtropical Asia, and North Africa [1, 2]. It can adapt to a variety of extreme types of weather, including hot and cold climates. In addition, is tolerant to saline soil. This plant is also used as a forage crop to feed animals and as green manure [3], a source of nectar, and a medicinal herb. This plant also aids in soil and water conservation [4, 5]. Before flowering, the young stems and leaves of the sweet yellow clover plant are easily eaten by animals, and it can be used as silage or converted to grass powder or hay [6]. It is highly nutritious with a crude protein content that is 4.6-fold higher than those of cereals, and the yield and ability of *Melilotus officinalis* to fix nitrogen are also better than those of alfalfa [7]. As a good source of nectar, it secretes large amounts of sugar and is an important source for honeybees to make honey [8]. The honey made from *Melilotus officinalis* is very influential throughout the world and is noted for its clear oral odor, regulation of sleep and metabolism, and enhancement of immunity [9]. It is also used as a medicinal herb and is rich in coumarin, which is an effective treatment for primary lymphedema and the lymphedema associated with radiation therapy, or surgery for breast cancer [10]. *Melilotus officinalis* can also be used to reduce swelling, inflammation, diuresis, and can treat various hemorrhoids and related diseases caused by them [11]. Moreover, it has been used to treat many cancers in recent years [12, 13].

The genomes of two species of *Melilotus* have been reported in recent years, including those of *Melilotus albus* and *Melilotus officinalis* [14, 15]. However, compared with other common legumes, there is limited knowledge on the structural and genetic information of *Melilotus officinalis*, particularly at the genomic level, which has substantially limited its breeding and improvement [16]. In this study, the genome information of *Melilotus officinalis* was obtained by combining Illumina (San Diego, CA, USA), PacBio (Pacific Biosciences of California, Menlo Park, CA, USA), HiFi (High fidelity), and Hi-C (high-throughput chromatin conformation capture) to fully understand the content of its genome and molecular evolutionary history. Hi-C technology was used to observe the collinearity between the chromosomes of *Melilotus officinalis* and its related species. This technique significantly improves the accuracy and sensitivity of evolutionary genetic research and enables the prediction of more robust patterns of genome structure [17]. The purpose of this study was to determine the positive selection of genes and the phylogenetic history of *Melilotus officinalis* when there were historical events and continuous changes in its geographical environment [18]. This study can help with subsequent genomic studies and provide a new research direction to analyze the evolutionary relationship between *Melilotus officinalis* and its close relatives.

## Results

### Genome survey, sequencing, and assembly

**Fig. 1:**
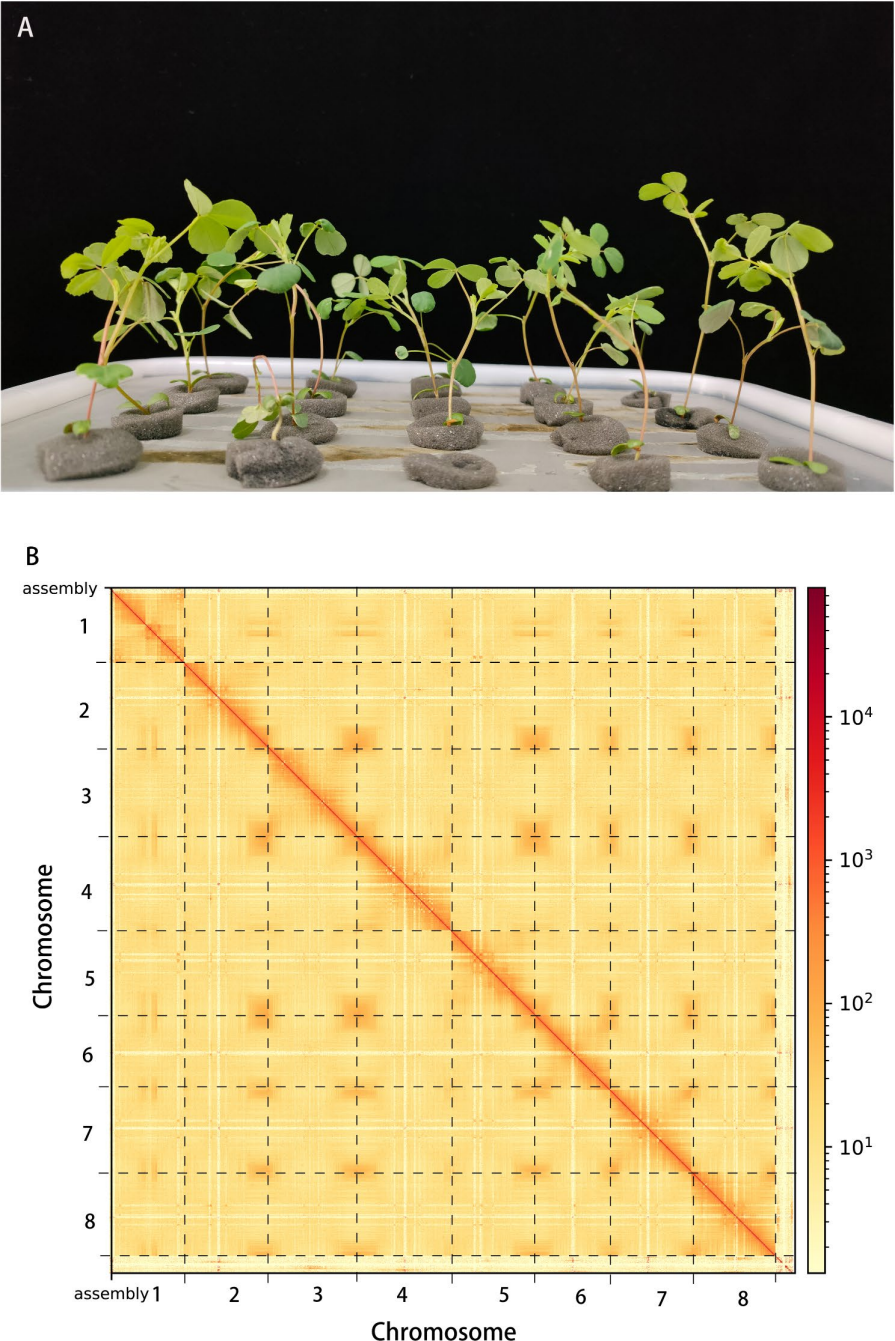
Plant morphology and Hi-C-assisted genome assembly of sweet yellow clover. (**A**) Phenotype of the sequenced sweet yellow clover plant. (**B**) Hi-C interaction heatmap showing 100-kb resolution super scaffolds. Hi-C, high-throughput chromatin confirmation system

In this study, samples were collected from Best corporation and sequenced using PacBio technology. Several genome parameters of *Melilotus officinalis* were obtained (Fig. 1A). The quality control results revealed that there were 83.6 Gbp of Illumina data with a GC content of approximately 34%. A total of 10,000 read sequences were randomly selected from the filtered clean reads and compared to the NT library through BLASTing, which mapped 97.85% of the sequences. A K-mer analysis can provide a general understanding of the genome before assembly [19]. This K-mer analysis indicated that the genome was 1,080 Mbp, and there were 67.3% repeat sequences and 1.76% heterozygous sequences. PacBio HiFi and Illumina technology were used to sequence the genome of *Melilotus officinalis* [20, 21]. Compared with traditional second-generation sequencing (NGS), the third-generation sequencing (TGS) technology developed by PacBio has the advantages of not requiring PCR amplification, producing long read lengths, and lacking a preference for GC [22, 23]. High-quality HiFi reads after CCS processing with 3 Mbp for HiFi reads, 16 kbp for N50, 57Gbp for base numbers, and a sequencing depth of 52X. A phased string graph was constructed using Hifiasm software, and contigs were generated according to the overlap map. The genome was 1,066 M, and it contained 492 contigs. Contig N50 was 5 Mbp. The largest contig size was 21 Mbp, and the average GC content was 35.38% (Table 1). The Illumina reads were compared with the DNA library to evaluate the quality and completeness of the assembly. The comparison indicated that 85.87% of the properly mapped reads were obtained. The completeness assessment of the assembled genomes was conducted by Benchmarking Universal Single-Copy Orthologs (BUSCOs) and the software TBLASTN, AUGUSTUS, and HMMER. The result was a complete BUSCOs of 96.4%, which showed that the genome assembly was high-quality [24, 25].

**Table 1:** Summary statistic for the *Melilotus officinalis* genome

	Assembly	This genome
Genome assembly	Estimated genome size	1080 Mbp
	Total length of assembly	1066 Mbp
	Number of contig	492
	Contig N50	5Mbp
	Largest contig	21Mbp
	Number of scaffolds	74
	Scaffold N50	130Mbp
	Chromosome coverage	97.34%
	GC content of genome	35.38%
Annotation		
Transposable elements	Total	761Mbp(71.35%)
	Retrotransposon	569Mbp(53.67%)
	DNA Transposon	62Mbp(5.90%)
Noncoding RNAs	rRNAs	5,168
	tRNAs	1,231
	miRNAs	416
	snRNAs	2,719
Gene models	Number of genes	47,873
	Mean gene length	4,041 bp
	Mean coding sequences	1,673 bp
	length	

**Table 2:** The information of annotated gene models per species for all the species

Organism	Number of genes	Mean coding sequences length(bp)	Exons per transcript	Mean exon length(bp)	Mean intro length(bp)
*Melilotus officinalis*	47,873	1,673	4.9	367	588
*Medicago truncatula*	38,823	1,342	4.8	278	540
*Trifolium medium*	28,496	560	1.7	321	499
*Vigna radiata*	30,878	1,337	5.1	261	530
*Trifolium subterraneum*	40,697	1,273	4.4	290	565

### Scaffold construction and curation

Hi-C is an extension of chromosome conformation capture (3 C) technology [26, 27]. Hi-C technology has become the primary choice for chromosome-level genome assembly and is widely applied in the assembly of animal and plant genomes [28, 29]. The Hi-C technique was used to obtain 136 Gb of data. A total of 97.34% of the initial assembly based on the PacBio data was scaffolded into eight chromosomes by the Hi-C data. The results of a Hi-C-assisted assembly revealed a genome size of 1,066 Mbp and a scaffold N50 of 130 Mbp [30]. After the Hi-C-assisted assembly had been completed, the inter-chromosomes and intra-chromosomes exchange interactions required calculation to determine if they were consistent with the principle of Hi-C genome assembly. The linkages within the chromosomes were much stronger than those between the chromosomes. Moreover, the linkages of chromosomes in a close physical location were much stronger than those in a distant physical location (Fig. 1B). These findings suggest that the assembly result was correct. Table 1 summarizes the information on assembly.

### Genome annotation

**Fig. 2:**
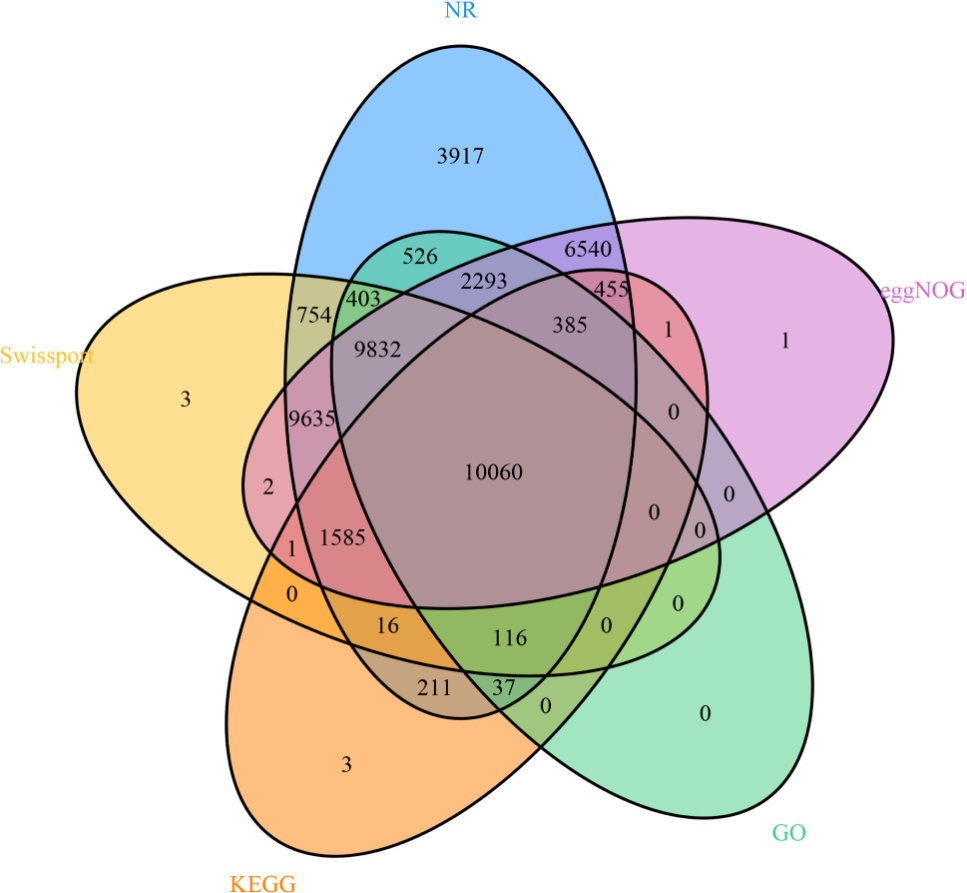
A Venn diagram that shows the overlap of the five major databases (NR, Swiss-Prot, eggNOG, GO, KEGG) that contain information from the annotation of gene function. GO, Gene Ontology; Kyoto Encyclopedia of Genes and Genomes

In this study, a total of 71.5% of the genome sequence was identified as repetitive, and it was 49.8% as long as the terminal repeat (LTR) transposable elements [31, 32]. There were 16.09% and 19.96% LTR retrotransposons of Copia and Gypsy, respectively, and there were 15,490 simple repeats in the assembled genome. There were 13 types of noncoding RNA (ncRNA) that totaled 10,016. We obtained 47,873 high confidence gene models by RNA-Seq assembly and gene prediction. The gene models were unevenly distributed on eight chromosomes. The average gene length was 4,041 bp, and each gene contained an average of 4.9 exons. The average lengths of coding sequences, exons, and introns were 1,673 bp, 367 bp, and 588 bp, respectively. We also compared *Melilotus officinalis* with four related species, including *Medicago truncatula* (MtrunA17r5.0-ANR from NCBI), zigzag clover (*Trifolium medium*) (ASM349008v1 from NCBI), mung bean (*Vigna radiata*) (ver6 from NCBI), and subterranean clover (*Trifolium subterraneum*) (TSUd_r1.1 from NCBI). *Melilotus officinalis* had the largest number of genes (47,873) and the longest average coding sequences at 1,673 bp. In contrast, *Trifolium medium* had the fewest genes (28,496). *T. medium* had the fewest average coding sequences and the average number of exons contained in each transcript among these five species. Although there was difference in the number of genes in the remaining three species, the lengths of their mean coding sequences were similar (Table 2). A functional annotation comparison analysis of the five databases annotated 46,776 genes, and the five databases collectively annotated 10,060 genes (Fig. 2). A total of 1,097 genes were not annotated (Table S1).

### Gene family and evolutionary analysis

**Fig. 3:**
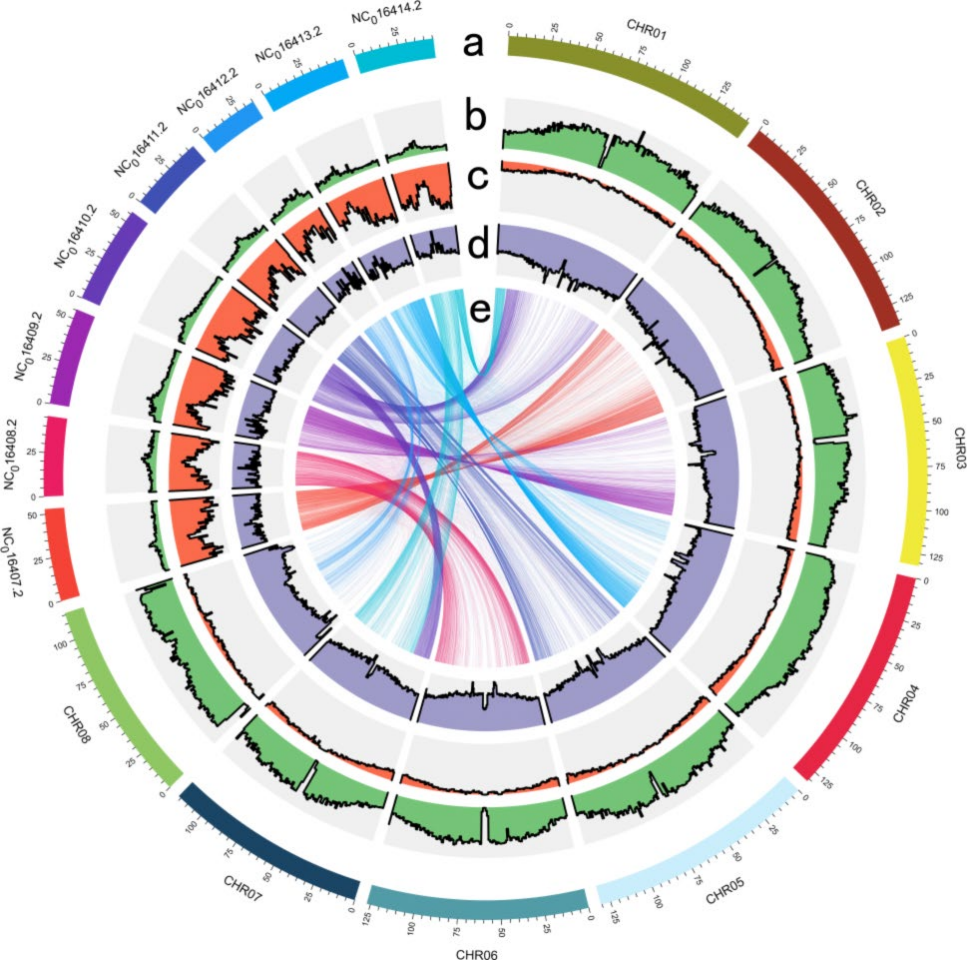
Feature of the *Medicago truncatula* and *Melilotus officinalis* genome. (**a**) Length of each pseudochromosome (Mbp). (**b**) Distribution of repetitive sequences (%). (**c**) Distribution of gene density (%). (**d**) Distribution of the GC content (%). (**e**) *Medicago_truncatula* and *Melilotus officinalis* synteny analysis; the beginning of NC represents the chromosome of *Medicago truncatula*, while the beginning of CHR represents the chromosome of *Melilotus officinalis*

**Fig. 4:**
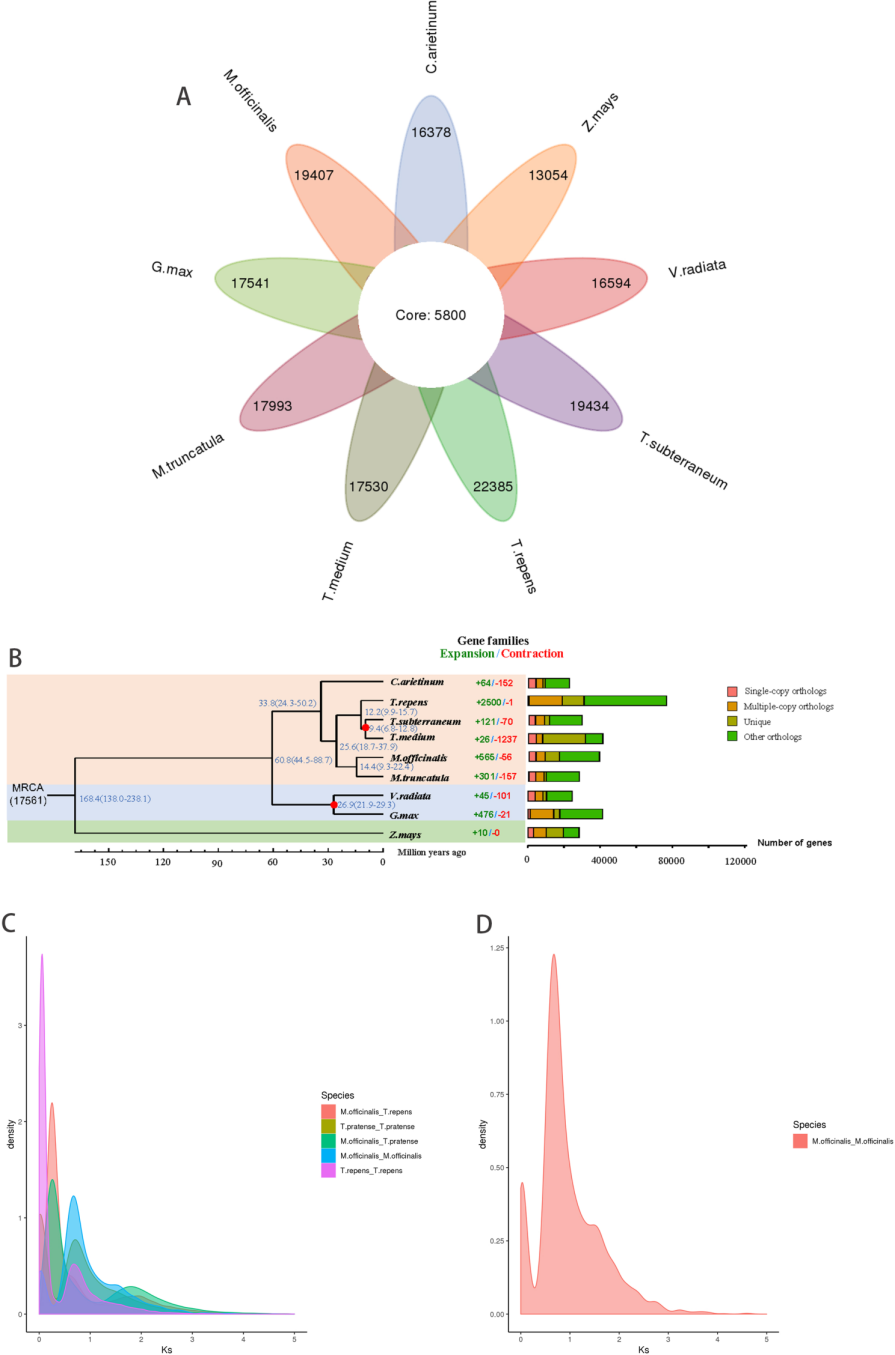
Gene family clustering and phylogenetic tree analyses of *Melilotus officinalis* and other representative plant genomes. (**A**) A Venn diagram of the number of shared gene families. (**B**) A phylogenetic tree based on shared single-copy gene families (left), gene family expansions and contractions among *Melilotus officinalis* and seven other species (middle), and Gene family clustering in *Melilotus officinalis* and seven other plant genomes (right). (**C**) Genome-wide replication Ks distribution map of *Melilotus officinalis* and its related species. (**D**) Genome-wide replication Ks analysis of *Melilotus officinalis*

The relationship between the eight chromosomes possessed by *Medicago truncatula* and *Melilotus officinalis* indicates that the chromosome synteny is conserved between the two species (Fig. 3) [33]. A gene family analysis of the genome of *Melilotus officinalis* and eight common species showed that 39,909 genomes were clustered in 25,207 gene families. There were 28,185 gene families in *T. repens*, and it shared 5,800 of these families among these several species (Fig. 4A). The analysis showed that it had expanded to 565 gene families and contracted 56 gene families during the course of evolution. A Gene Ontology (GO) analysis showed that the expanded gene family was related to response to stimulus, nucleotide binding, and small molecule binding. Gene families with biological process are the most abundant (Table S2). These gene families could be involved in plant metabolic processes that are involved in the resistance of plants to stress, which enables the plants to more effectively adapt to changes in the environment. A phylogenetic tree was constructed based on 3,870 single-copy homologous genes, with maize (v. 5.0 from NCBI) as the outgroup. *Melilotus officinalis* clustered with soybean (v. 4.0 from NCBI), chickpea (ASM33114v1 from NCBI), mung bean (v. 6 from NCBI), subterranean clover (TSUd_r1.1 from NCBI), *Medicago truncatula* (MtrunA17r5.0-ANR from NCBI), white clover (AgR_To_v5 from NCBI) and zigzag clover (ASM349008v1 from NCBI) to form a monophyletic group. Single-copy genes of each species were selected as reference markers for the species with incomplete evolutionary studies. The closest relationship was between sweet yellow clover and *Medicago truncatula*, with an estimated time of divergence of approximately 14.4 million years ago (Fig. 4B). Whole-genome duplication (WGD) events are an important indicator of plant evolution and a driving force for the adaptation of plants to various environments [34, 35]. The evolutionary history of the yellow sweet clover plant can be understood by studying the number of synonymous substitutions that occurred at each synonymous site in its genome [36]. The data suggest that both sweet yellow clover and white clover in the self-comparisons had peaks at approximately 0.75 (Fig. 4C) [37, 38]. In addition, the WGD event occurred when the KS value of sweet yellow clover was 0.75 (Fig. 4D).

## Discussion

The genomic information of leguminous plants with good agronomic traits is very important for the study of genomics and functional omics [39, 40]. This is not the first report of the assembly of *Melilotus officinalis* since a chromosome-scale assembly of *Melilotus officinalis* has been reported [14, 41]. *Melilotus officinalis* is not only an excellent forage crop that is highly valuable nutritionally; it is also highly valuable medicinally. Although the genome of *Melilotus officinalis* has been published, the differences in sequence were not the same as that observed in this study. This study can enrich the genetic information database of *Melilotus officinalis* and lay a foundation for the further excavation of special genetic markers of *Melilotus officinalis*. For example, if a pan-genome analysis is conducted, it is necessary to sequence as many representative samples of the same plant as possible. Simultaneously, this study adopted the current mainstream Hi-Fi sequencing technology to improve the assembly quality and compensate for gaps in the study of the characteristics of *Melilotus* species. This study enriches the knowledge about legumes and provides research experience for the subsequent study of *Melilotus officinalis.* In this study, a K-mer analysis was used to estimate the genome size, heterozygosity, and repeat sequence ratio, which was the same method utilized in the recently published genome. The K-mer analysis showed that the *Melilotus officinalis* genome was heterozygous (1.76%), highly repetitive (67.3%), and comprised a large and complex genome. The result of K-mer analysis was the same as the published genome with heterozygosity (0.06%) and repetition (71.94%). The genome size was estimated to be 1080 M, which was similar to the genome of *Melilotus officinalis* (1.09 Gb) [14]. Compared with the previously published genome of *Melilotus officinalis*, both the latest PacBio third generation HiFi assembly and sequencing strategies were used to obtain the genome information. The previously published genome assembly of the *Melilotus officinalis* size was 976.27 M (contig N50 = 7.02 Mbp, scaffold N50 = 125 Mbp, number of contigs = 295) compared with 1,066 M (contig N50 = 5Mbp, scaffold N50 = 130 Mbp, number of contigs = 492) that was reported this study, which indicated that the quality had significantly improved (Table 1). The assembled genome had an average GC content of 35.38%, which was close to that of the previously assembled *Melilotus officinalis* genome (35.50%). The assembled genome at the chromosome level covered 97.34%, which was also close to the previously published genome assembly of *Melilotus officinalis.* BUSCOs were used to compare the published genomes, which enabled an assessment of the integrity of the genomes, and the results showed that both genomes were fully assembled. Compared to the result of the Hi-C-assisted assembly of the published assembly genome, there were more clean reads and clean bases than in the published assembly genome. The number of unique mapped read pairs was also significantly higher than that of the published assembly genome. After Hi-C scaffolding, the genome annotation revealed 47,873 high-confidence gene models, which was close to the published assembled genome that identified 50,022 annotated genes. Compared with the published assembly genome, the process used to predict the repeat sequences was basically the same, but MITE Hunter v1.0, LTR Finder v1.07 and LTR harvest were used to predict these repeat sequences, which had not been reported in the published assembly genome (Table S3). The results of the prediction of noncoding RNAs revealed that were 5,168 rRNAs, 1,231 tRNAs, 416 miRNAs, and 2,719 snRNAs compared with the published assembly genome (rRNAs = 673, tRNAs = 934, miRNA = 125, snRNAs = 244). The prediction of noncoding RNAs in this study resulted in better results than those in the published assembly genome (Table 1). The mean coding sequence length of our assembly genome was 1,673 bp, which was slightly higher than the mean coding sequence length of the published genome (1290.3 bp). However, there were fewer mean exon lengths and mean intro lengths than in the published assembly genome (Table 2). In this study, a Venn analysis was performed on the five major databases to obtain the results of gene function annotation, while a Venn analysis was not performed in the published assembly genome (Fig. 2). The recently published assembly genome used OrthoFinder to analyze clustering of the protein family to compare *Melilotus officinalis* with this parameter in seven other common legumes. These analyses revealed that 891 gene families were unique to *Melilotus officinalis*, and 7,596 gene families were shared by eight legumes. However, in this study, OrthoMCL was used to perform a cluster analysis and compared *Melilotus officinalis* with other eight common legumes. The results showed that *Melilotus officinalis* had 19,407 gene families, and nine legumes shared 5800 gene families. The different results may be owing to comparisons of different species or the use of different software for the analysis. The published assembly genome revealed that there were 635 significantly expanded gene families and 729 significantly contracted gene families in *Melilotus officinalis.* In this study, expansion and contraction of the gene families showed that *Melilotus officinalis* expanded by 565 gene families and shrank by 56 gene families. The expanded genes were mainly involved in proteolysis, peptidase activity and defense response (Figure S1). The contracted genes were mainly involved in response to stress, nuleotide binding, small molecule binding and response to stimulus (Figure S2). These results revealed the expanded and contracted genes affected the stress resistance of *Melilotus officinalis*. These findings provide a basis for subsequent research on molecular breeding. The evolutionary history of *Melilotus officinalis* has been less well-studied. A genome collinearity analysis revealed that *Melilotus officinalis* and *Medicago truncatula* had a high degree of genome collinearity. The WGD events revealed that sweet yellow clover diverged after mung bean, maize (*Zea mays*), soybean (*Glycine mas*), and chickpea (*Cicer ariantum*) and before subterranean clover, white clover, and zigzag clover. Sweet yellow clover and *Medicago truncatula* basically differentiated at the same rate. The ancestors of these species were similar to those of sweet yellow clover. In this study, the genomes of sweet yellow clover and related species were compared at the genomic level. The structural genomic features and gene function of sweet yellow clover were explained by collinear analysis. In addition, a phylogenetic tree construction and analysis, cluster analysis of gene protein families, and a gene contraction and expansion analysis of sweet yellow clover were also conducted. There were also limitations to this study. First, a Hi-C assisted genome assembly was adopted in this study, and the latest T2T genome assembly can complete telomere to telomere assembly, which can improve the quality of genome assembly [42]. Secondly, genomic information is the basis of research function, but the genetic mechanism of related phenotype shape is very complex. How to effectively conduct multi-omics research is also a problem [43]. The future research direction of this area should be to sequence the transcriptome and metabolome of sweet yellow clover, which can facilitate a better understanding of its biological processes [15]. Perhaps single-cell sequencing could be conducted, and single-cell sequencing can help to better understand the regulatory mechanisms of the gene and study the molecular mechanisms at the single-cell level [44]. The genomic information of sweet yellow clover will help to understand the evolution of leguminous plants. The medicinal value of sweet yellow clover also merits study because it produces coumarin [45, 46].

## Conclusions

This study reported the third generation Hi-Fi assembly of the PacBio platform, a genome with high coverage and higher completeness in published genomes. Moreover, this study can provide insight into the evolution of vegetation and provide a genetic gene pool for subsequent studies.

## Materials and methods

### DNA isolation and sequencing

A DNA secure kit (TianGen, Beijing, China) was used to isolate genomic DNA from the leaves of sweet yellow clover in the Grassland Agri-Husbandry Research Center, College of Grassland Science, Qingdao University (Qingdao, China). The DNA was sequenced by Berry Hekang (Beijing, China) using the PacBio third generation HiFi assembly sequencing platform [20]. First, the quality of samples was tested. The libraries were established to be subjected to PE sequencing using Illumina NovaSeq. Raw reads that contained adapters, duplicates, and low sequence quality were first filtered and then followed by a random selection of 10,000 of the reads for comparison with the NT (Nucleotide Sequence Database) [47] library using BLAST v. 2.12.0 [48]. No significant external contamination was detected. The K-mer counting method was used to estimate the genome size. Clean reads from the Illumina library were used to estimate the genome size using k-mer = 23 analysis by Jellyfish v1.1.11 (Table S3) [49]. The formula for estimating the size of the sweet clover genome is as follows:

$$ G\hspace{0.17em}=\hspace{0.17em}{K}_{number}/{K}_{depth}$$

where *K*_depth_ is the expected depth of the k-mers.

### Genome assembly and quality evaluation

A NanoDrop 2000 spectrophotometer (Thermo Fisher Scientific, Waltham, MA, USA) was used to determine the quality of the genomic DNA. The degree of DNA degradation and the presence of RNA contamination were analyzed by pulsed field electrophoresis and Fragment Analyzer capillary electrophoresis [50]. The purified genome was subsequently constructed into a SMRTbell library and then sequenced using PacBio SMRT technology [51, 52]. An Agilent 2100 bioanalyzer (Agilent Technologies, Santa Clara, CA, USA) was used to determine the size of library. The data obtained was filtered and then processed using the SMRTLink v. 8.0 software for CCS processing. Hifiasm v. 0.15.2 software was used for assembly, followed by de-hybridization of the contig sequence using the purge-dups v. 1.2.3 software (Table S3) [21, 53]. A single-copy orthologous gene library eudicots_odb10 [54] that was combined using TBLASTN [55], AUGUSTUS v3.2.2 [56], and HMMER [57] software was finally used to evaluate the integrity of the assembled genome [58] (Table S3).

### Hi-C data analysis and chromosome construction

Leaf cells (100 mg) of *Melilotus officinalis* were first treated with the cell crosslinking agent paraformaldehyde for 15 min. The DNA and protein were crosslinked; the conformation of DNA was fixed, and glycine was added to prevent the chromatin from crosslinking. The treated leaf tissue was collected and frozen in liquid nitrogen. The leaf tissue was then ground in preparation for subsequent DNA extraction [59]. Biotin was added at the time of end repair to label the oligonucleotide ends. The extracted DNA was subsequently resolved into 350 bp fragments using Covaris and sequenced in the PE150 mode using an Illumina NovaSeq 6000 sequencing platform [60]. The raw reads obtained by sequencing were not all effective, and these raw reads needed to be finely filtered to obtain effective high-quality clean reads. A total of 10,000 read sequences were randomly selected from these filtered clean reads, and their contamination was assessed by alignment to the NT library using BLAST v. 2.12.0 (Table S3) [61]. The Hi-C data were aligned to the preliminary assembled genome using Juicer v. 1.6.2 (Table S3) [28]; the results were filtered and corrected, and then the Hi-C library results were analyzed using 3D DNA 180,922 software (Table S3) [62, 63]. The scaffold of *Melilotus officinalis* was obtained at the chromosome level.

### Repeat annotation and gene annotation

The predicted repeats and known repeats in the genome were first masked using Repeat Masker v. 4.1.0 (Table S3) [64, 65]. The repeats were again predicted using MITE Hunter v. 1.0, LTR harvest, LTR Finder v. 1.07, LTR retriever v. 2.8.2, and Repeat Modeler v. 2.0 (Table S3) [66]. The MITEs and LTR transposable elements were then identified using structural prediction methods [67, 68]. Class II transposable element MITEs, as well as nonautonomous transposable elements < 2 kb long, were searched from the genome using MITE-Hunter v. 1.0, and the analysis was performed using the software default parameters to enable the prediction of MITEs [69, 70]. The prediction of LTR transposable elements required the use of LTR harvest and LTR Finder v. 1.07 [71]. First, an LTR harvest was used to predict the LTR-RT in the genome using the parameters of the software is-similar 90-vic 10-seed 20-seqids yes-minlenltr 100-maxlenltr 7000-mintsd 4-maxtsd 6-motif TGCA-motifmis 1 [50]. The LTR-RT was predicted using the LTR-Finder v. 1.07 with the following software parameters: -D 15,000-d 1000-L 7000-l 100-p 20-C-M 0.9 [72] (Table S3). The repeats in masked genomes were identified *de novo* using RepeatModeler v. 2.0 with the following software parameters: -engine ncbi-pa 60 [73]. RepeatMasker v. 4.1.0 was then used to block the repetitive sequences in the genome, and the software utilized the following parameters: -s-nolow-norna-gff-engine ncbi-parallel 20 [74] (Table S3). The ab initio prediction for tRNA was performed using the software tRNAscan-SE v. 2.0, and rRNA and other types of ncRNA were searched by their similarity when aligned with the Rfam database (https://ftp.ebi.ac.uk/pub/databases/Rfam/14.1/) [75, 76] (Table S3). All the repetitive regions except tandem repeats were soft-masked to annotate the proteins that encoded genes [77, 78]. GeMoMa-1.6.1 was used to compare the protein sequences of related species with the assembled genomes. These comparisons were then combined with the comparison of RNA data and assembly results to obtain exon and intron boundary information and improve the prediction accuracy [79, 80]. A comprehensive transcriptome database was constructed using PASA (v. 2.0.1). The gene structure was predicted with AUGUSTUS v3.2.2 combined with the RNA-Seq data, SNAP v6.0 and GlimmerHMM v3.0.4 [81, 82] (Table S3). The RNA-seq data was used to annotate the gene structure to optimize the accuracy of gene structure annotation and provide a reliable training set for the *de novo* prediction software. The parameters were trained with the training set, and the Scaffold with the masked repeat sequence was utilized [83, 84] The predictions obtained using these packages were combined using EVIdenceModeler (EVM) r2012-06-25 [68] (Table S3), and then 36,511 genes were retrieved and functionally annotated by BLAST searches against databases, including NR (http://ftp.ncbi.nlm.nih.gov/blast/db/) [85], Swiss-Prot (http://ftp.ebi.ac.uk/pub/databases/uniprot/knowledgebase/uniprot_sprot.fasta.gz) [86], eggNOG (http://eggnog6.embl.de.) [87], Gene Ontology (GO) (http://geneontology.org/) and the Kyoto Encyclopedia of Genes and Genomes (KEGG) (http://www.genome.jp/kegg/) [88]. A Venn diagram of the five major databases was then performed to obtain more accurate information on the functional annotation of the genes [89].

### Comparative analysis

Genomic collinearity was analyzed on *Melilotus officinalis* and its related species *Medicago_truncatula* using MuMMER v. 4.1 software, The parameters of the software were as follows: nucmer-g 1000-c 90-l 200 [90] (Table S3). Nine protein families were identified by OrthoMCL cluster analysis, including chickpea, soybean, *M. truncatula*, zigzag clover, white clover [*T. repens*], subterranean clover, mung bean, maize, and *Melilotus officinalis*) [91]. An all-vs-all BLAST alignment of all the sequences of *Melilotus officinalis* genes that encode proteins (with 1e^− 5^ as the default e-value) was first performed and then followed by a calculation of the sequence similarity [92, 93]. The Markov clustering algorithm was then used for cluster analysis with an expansion coefficient of 1.5 to obtain the clustering results for the protein families [94, 95]. Owing to a lack of research on the evolution of *Melilotus officinalis*, selected species single-copy genes were used as a reference marker to select the four degenerate sites to construct a supergene using the MAFFT v. 7.310 software for multiple sequence alignment [96, 97] (Table S3). The most suitable base substitution model was selected with RAxML software that was based on the maximum likelihood (ML) species phylogenetic tree. MCMCtree was used from the PAML v. 4.9e package based on a single copy gene family (parameter: burn-in = 5,000,000, sample-number = 1,000,000, and sample-frequency = 50) [98] (Table S3). The time of differentiation was estimated. Time calibration points (correction points) were from the Timetree website [99]. The gene family was then analyzed using CAFE v. 3.1 software and GO functional enrichment analysis for the genes in these families (Table S3) [100–102]. A branch-site model can detect positive selection that occurs in a particular clade and only affects a portion of the locus. The one-to-one orthologous proteins were selected from *Melilotus officinalis* and its related species, and the homologous protein sequences were aligned using the default parameters of PRANK software [103, 104].

gBlocks were used to filter the alignment results with the parameters -t = c-e=. For ft-b4 = 5-d = y, the CODEML test in PAML v. 4.9e was located in a specific clade and only affected positive selection at certain sites. It was corrected for multiple hypothesis testing using the Chi2 program in PAML v. 4.9e. The main parameter was 2 degrees of freedom [105] (Table S3).

The WGD events were detected using the duplicate age distribution method. The longest protein sequences of genes in the *Melilotus officinalis* genome were then aligned using BLASTP. The alignment was filtered using the DAG chainer, and the synonymous substitution rate was calculated using the Yn 00 tool in the PAML v. 4.9e software package [106]. A map of density distribution based on the Ks values of all the paralog gene pairs and the Ks values of orthologous gene pairs between the genomes of *Melilotus officinalis*, white clover, and other related species was then drawn using MATLAB [107] (Table S3).

### Electronic supplementary material

Below is the link to the electronic supplementary material.

Supplementary Material 1

Supplementary Material 2

Supplementary Material 3

Supplementary Material 4

Supplementary Material 5

## Data Availability

All raw data were submitted in NCBI Database (SRR23985850, SRR23985849, SRR23985851, SRR23985848). The details of software used are in Table S3. and the genome assembly and annotation were uploaded in the dedicated public repositories (assembly of *Melilotus officinalis*: DOI: 10.6084/m9.figshare.23590107, genome annotation of Melilotus officinalis: DOI: 10.6084/m9.figshare.23590161).
