# Supplementary figures and images for: Genome assembly of Melilotus officinalis provides a new reference genome for functional genomics
Source: BMC Genom Data. 2024 Apr 18;25:37. doi: 10.1186/s12863-024-01224-y (PMC11025269; doi:10.1186/s12863-024-01224-y)

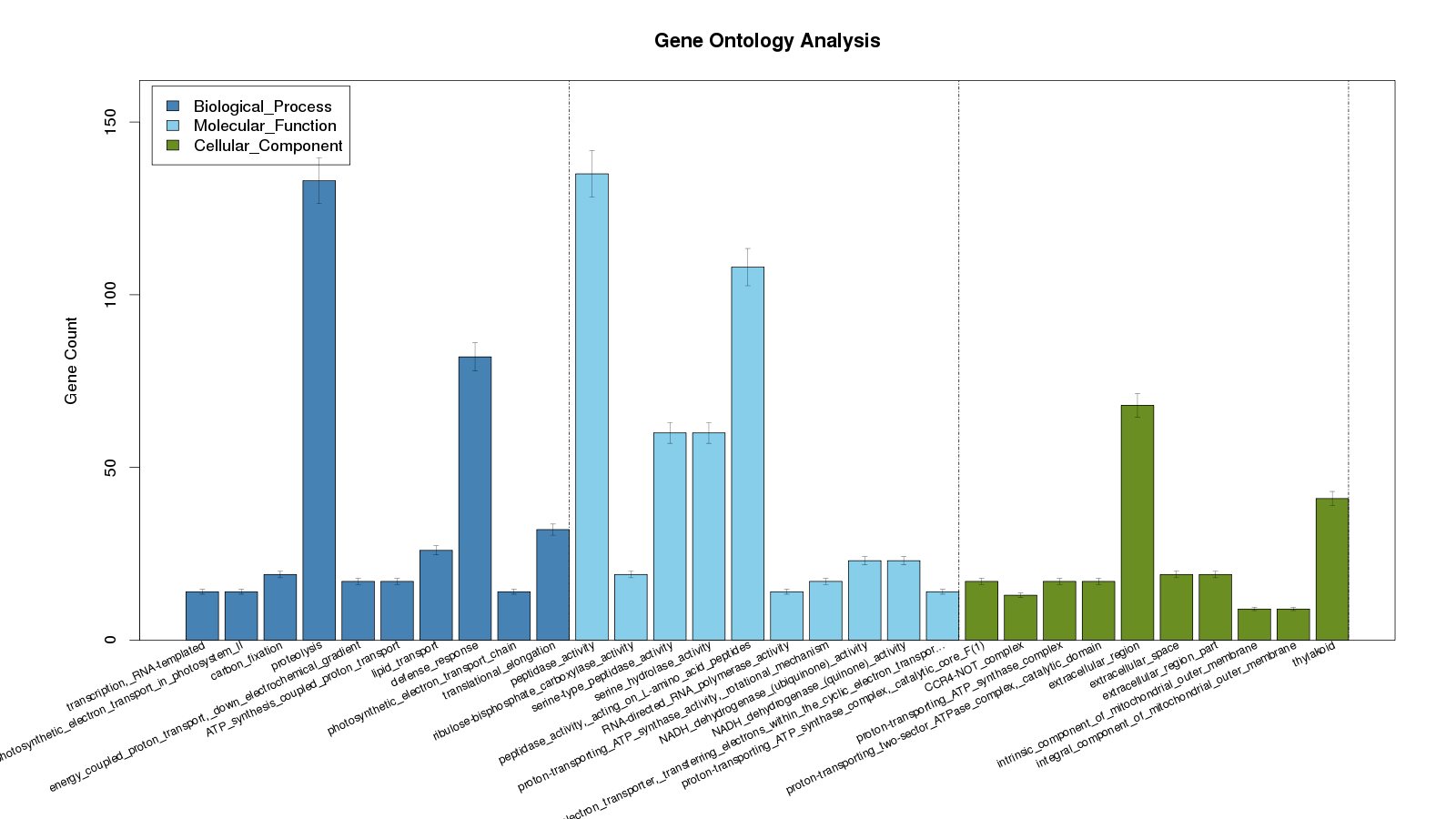

Supplement: Supplementary file 4 — Supplementary Material 4 [file 12863_2024_1224_MOESM4_ESM.png]

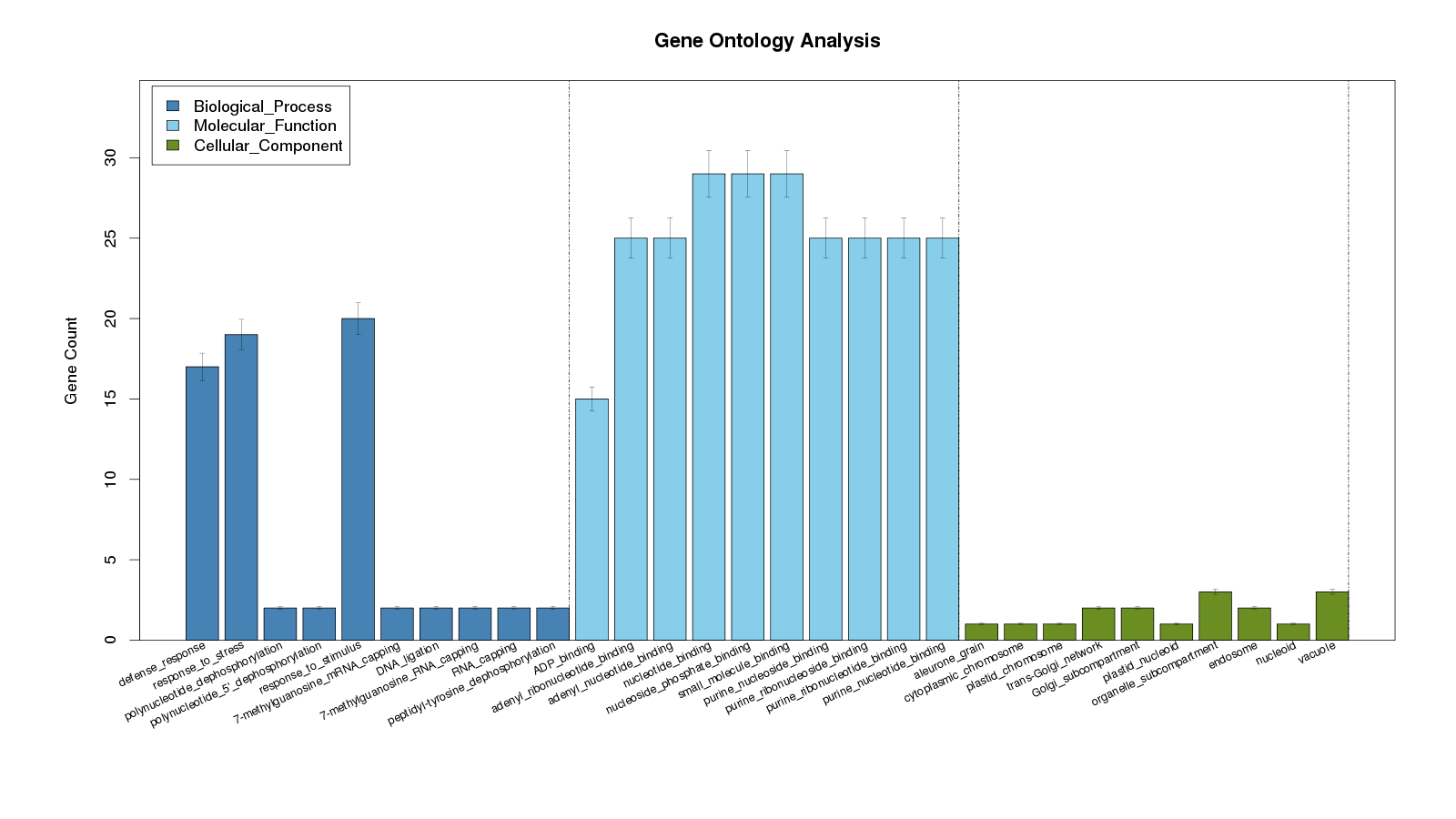

Supplement: Supplementary file 5 — Supplementary Material 5 [file 12863_2024_1224_MOESM5_ESM.png]
